# A one-day surgical-skill training course for medical students’ improved surgical skills and increased interest in surgery as a career

**DOI:** 10.1186/s12909-017-1106-x

**Published:** 2017-12-28

**Authors:** Ho Seok Seo, Yong Hwa Eom, Min Ki Kim, Young-Min Kim, Byung Joo Song, Kyo Young Song

**Affiliations:** 1 Department of Surgery, Seoul St. Mary’s Hospital, College of Medicine, The Catholic University of Korea, 222 Banpo-daero, Seocho-gu, Seoul, South Korea; 2 Department of Emergency Medicine, College of Medicine, Seoul St. Mary’s Hospital, The Catholic University of Korea, 222 Banpo-daero, Seocho-gu, Seoul, South Korea; 3 START Center for Medical Simulation, College of Medicine, The Catholic University of Korea, Songeui-building, 222 Banpo-daero, Seocho-gu, Seoul, South Korea

**Keywords:** Surgical skill, Intensive training, Medical students

## Abstract

**Background:**

Despite many high-quality programs in basic surgical-skill education, the surgical skill of junior doctors varies widely. This, together with the waning interest in surgery as a career among medical students, is a serious issue confronted by hospitals and healthcare systems worldwide. We, therefore, developed and implemented an intensive one-day surgical-skill training course for two purposes; it would improve surgical skills and increase interest in surgery among medical students.

**Methods:**

The surgical-skill training program is named Surgical Skill Weekend (SSW) and it includes hands-on training sessions for surgical-suturing techniques and advanced surgical procedures (i.e. laparoscopic and robot-assisted surgery), hybrid simulation sessions, and an operating-room session where aforementioned sessions are all put together. By the end of the program, students’ improvements in surgical-suturing skills were assessed by experts in a form of checklist, and changes in the interest in a surgical career, if there were any, were answered by the students who participated in the program.

**Results:**

A total of ninety-one (91) medical students participated in the 2015 and 2016 SSW courses. Their overall satisfaction level with the course was very high (Very satisfied: 78%, Quite satisfied: 22%). All of the participant’s surgical-suturing skills significantly improved (median score range: 14–20, *P < 0.05*) and their interest in a surgical career increased significantly (from 56% to 81%, *P < 0.05*) by completing the program.

**Conclusions:**

An intensive and comprehensive surgical-skill training program for medical students can not only improve surgical-suturing skills but also increase interest in surgery as a career.

**Electronic supplementary material:**

The online version of this article (10.1186/s12909-017-1106-x) contains supplementary material, which is available to authorized users.

## Background

Historically, medical students have been instructed and trained in surgical skills in the operating room under the supervision of senior surgeons [1]. Although such apprenticeship training was once appropriate [2], in more recent decades the lack of standardization has emerged as a serious problem. These days, most surgeons consider the development of basic surgical-skill courses and standardized assessment tools are necessary in medical students’ education. For this reason, various types of training modules and assessment tools have been developed for the purpose of training of medical students or surgical residents. There have been a number of studies regarding standardization of surgical training protocols. Martin et al. assessed surgical residents using the Objective Structured Assessment of Technical Skill (OSATS) tool and found that it could reliably and validly assess surgical skills [3].

In Korea, clinical-skill tests have been part of the Korean Medical License Examination (KMLE) since 2009. The KMLE includes two types of such assessment: a clinical performance examination (CPX) using standardized patients (SPs) and an objective-structured clinical examination (OSCE) using partial-task trainers and SPs [4]. Despite the introduction of clinical-skill tests, surgical-skill performance varies widely among students. This is partly due to the fact that training programs are still not standardized among institutions and, in some cases, such training programs vary from the real practice.

Surgery, from long time ago, has been one of the most prestigious and important medical department. More recently, however, it has been regarded by medical students as a 3D (difficult, dirty, and dangerous) job [5]. In the United States and Europe, the number of applicants for surgery residency has decreased, and many residents are giving up their training before completion [6, 7]. Korea is not so much different. According to the Ministry of Health and Welfare, the number of applicants for surgery residency in 2008 reached below 62% of the number limit; moreover, the number of residents giving up their training has risen by more than 12%. The decreased number of applicants results in laboriousness during the training. This again results in the decrease of applicants, making it a vicious cycle. To end this cycle, introducing surgery and making surgery attractive is important [8]. Certainly, a systematic and comprehensive student training program that can simulate actual surgical work is urgently needed.

Some years ago (2011), we developed a systemic, comprehensive, and intensive one-day~~ known as Surgical Skill Weekend (SSW), and recently updated it. The program mainly focuses on the systematic training for basic surgical-suturing techniques including simple suturing and vertical mattress suturing. Students are instructed initially using a simple artificial skin, and subsequently undergo hybrid-simulation-based training with SPs or patient simulators. Additionally, to increase students’ interest in surgery, animal-intestinal anastomosis as well as laparoscopic- or robot-assisted surgery simulators training are incorporated into the program. The goals of the SSW program are not only to educate the surgical skill, but also to introduce the culture of surgery to the medical students.

The aim of this study was to determine whether a one-day intensive surgical-skill training course can improve medical students’ surgical-suturing skill and increase their interest in surgery as a career.

## Methods

### Participants

Medical students who had taken the 2015 or 2016 SSW course in their first, second, third or fourth year were enrolled in the study. The SSW, which was promoted to all medical colleges in Korea through internet articles, social network services (SNS) and pamphlets, has been held annually since 2011 at Seoul St. Mary’s Hospital, Seoul, Korea. For consistently high program quality, the enrollment is limited annually to fewer than 50 students, and they are divided into 12 groups (each with a tutor-to-student ratio of 1:3 or 1:4). This study was approved by the Institutional Review Board of the Ethics Committee of the College of Medicine, The Catholic University of Korea (KC16QISI0775).

### Program

**Fig. 1:**
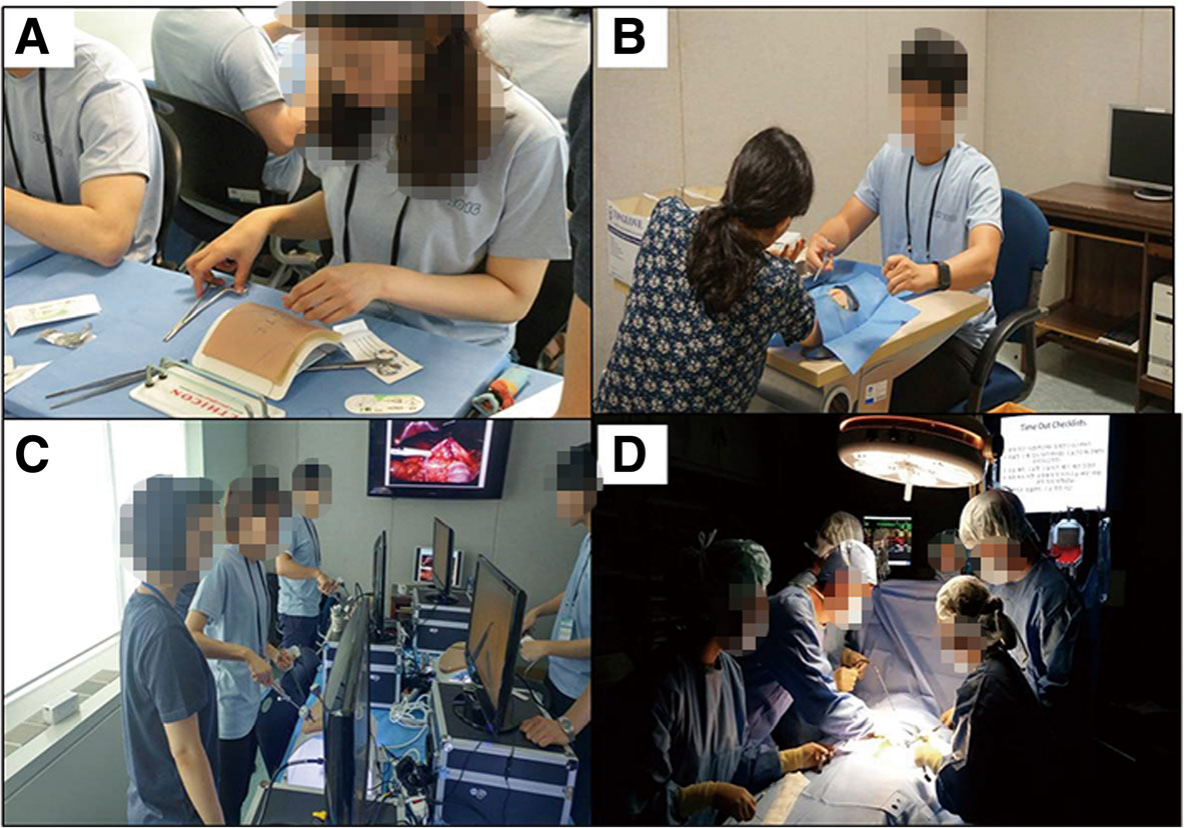
Four sessions of program: **a** Basic surgical technique session **b** Hybrid simulation session **c** Laparoscopic or robot-assisted surgery session **d** Putting-it-all-together operating-room session

The program director and the tutors had gone through seven meetings and rehearsals for six months to determine the composition of the program. Finally, four sessions were selected (Fig. 1). The first session, “basic surgical technique,” includes standardized procedures such as simple and vertical mattress suturing of artificial skin, surgical tie, and animal-intestinal anastomosis. In the second session, “hybrid simulation,” students play the roles of physicians in various fields and treat SPs who have artificial skins on their forearms. The third session, “laparoscopic or robot-assisted surgery”, requires students to perform the laparoscopic suturing technique and undergo training for robot-assisted surgery, both of which are experienced only rarely in the course of an actual clinical practice. The fourth session was a “putting-it-all-together” session. A room was designed to be a realistic operating room, and the tutor acted as an anesthesiologist and a scrub nurse assisted the students. Students performed, in turn, scrubbing, gowning, painting, draping, incision, and suturing to a mannequin as the operator. First- and second-year students attended the basic surgical technique session, the basic laparoscopy training session and the hybrid simulation session; third- and fourth-year students, meanwhile, attended the basic surgical technique session, the advanced laparoscopic and robot-assisted surgery session and the putting-it-all-together operating-room session.

### Data collection

Surgical-suturing skill tests were performed before and after the training. All of the participants were assessed according to a standardized 10-item checklist. The check lists were adopted from those in the OSCE in the KMLE for the standardization. The checklist included basic suturing skills such as correctness of suture materials or instruments, handling of tissue and instruments, and the time taken in the procedure. The pre-program test was performed after the participants attended a didactic lecture and watched a video clip. After the completion of the course, they took the same test again. Both tests were assessed by the tutors, who had been trained with the checklist and with a video demonstrating the standard technique for each suture. If the participants performed correctly, it was scored as 2 points, if incorrectly performed 1 point, and if not performed at all, 0 point (Table 1).

**Table 1:** Pre-to-post-program evaluation form

Evaluation items	Correct (2)	Incorrect (1)	Undone (0)
Using proper suture materials			
Using proper tissue forceps			
Using proper instruments (needle holder)			
Holding 2/3~3/4 point from the needle tip with the needle holder			
Keeping right angle during insertion and extraction of needle into and from skin			
Using forceps suitably during needle insertion into skin			
Knotting more than two times and not allowing knots to become loose			
Maintaining tension after finishing suturing			
Eversion of skin edges of even thickness			
Finishing procedure in time (15 minutes)			
Total	/20		
Tutor (sign)			

After the program, a survey on the SSW and participants’ interest in pursuing surgery as a career was conducted. We then compared the results of the suturing skill tests and the survey data to evaluate the impact of the program.

### Statistical analysis

Based on a normal data distribution, descriptive data were presented as frequencies and percentages, means and SD or medians and ranges. Chi-square tests, *t*-tests, Mann-Whitney tests or Wilcoxon signed rank tests were used to compare the pre- and post-program test scores with the survey as appropriate. Statistical analyses were performed with SPSS ver. 21.0 software (SPSS Inc., Chicago, IL, USA). Values of *p* < 0.05 were considered statistically significant.

## Results

A total of 91 students participated in the SSW program, including 46 in 2015 and 45 in 2016. There were 19 first-year, 33 s-year, 34 third-year, and 5 fourth-year students (Additional file 1).

The survey included questions on why the students decided to participate in the program, which of the sessions they found useful or insufficient, their level of satisfaction, and among others, their change in interest in a surgical career. Most of the applicants had come to the course through the introduction from senior, former SSW participants or after having read a promotional pamphlet on the internet. The most useful sessions were animal-intestinal anastomosis and hybrid simulation session. The results on the participants’ overall satisfaction with the course were as follows: 78% very satisfied and 22% quite satisfied. There were no participants who answered moderate satisfaction, dissatisfaction, or “not sure”.

All of the participants’ skills were assessed by the checklist before and after the course. The pre-course score distribution ranged from 6 to 20, and the median score was 14. After the course, 5 students obtained the lowest score of 17, and 86 students scored 18 or more. The median score was 20. The 14-to-20 improvement in median score was statistically significant according to the Wilcoxon signed rank test (Table 2).

**Table 2:** Score distributions before and after program

Score (*n* = 91)	Before the program	After the program	Improvement	*p-value*
Median [Range]	14 [6–20]	20 [17–20]		0.000
Mean ± SD	14.0 ± 3.111	19.4 ± 0.879	5.4 ± 2.929	0.000
Distribution, n (%)				
20	4 (4.4)	54 (59.3)		
19	3 (3.3)	23 (25.3)		
18	5 (5.5)	9 (9.9)		
17	4 (4.4)	5 (5.5)		
16	15 (16.5)			
15	9 (9.9)			
14	12 (13.2)			
13	12 (13.2)			
12	10 (11.0)			
11	3 (3.3)			
10	4 (4.4)			
9	7 (7.7)			
8	1 (1.1)			
7	1 (1.1)			
6	1 (1.1)			

The participants were divided into two subgroups: the “low year” group comprised students who were in their first or second year and the “high year” group in their third or fourth year. The students in the “low year” group showed a significantly greater post-program improvement in suturing skills than the “high year” group did (Table 3).

**Table 3:** Score-improvement comparison by year

Score (median)	Low year (*n* = 52)	High year (*n* = 39)	*p-value*
Before program	13	16	0.000
After program	20	20	0.131
Improvement	6	4	0.000

Low year: first- and second-year students; High year: third- and fourth-year students

The results of the pre-to-post-course change in the interest in a surgical career are shown in Table 4. As indicated, the number of students willing to apply to a surgery residency program increased from 51 to 74; the number of those unwilling to do so decreased from 11 to 1.

**Table 4:** Interest in applying to Department of Surgery

Interest in surgical career (n = 91)	Before program	After program
Yes	51 (56.0%)	74 (81.3%)
No	11 (12.11%)	1 (1.1%)
Do not know	29 (31.9%)	16 (17.6%)

## Discussion

The most important elements of surgical-skill training are realistic simulation of procedures and an overall systematic approach. Several studies have established that simulation-based medical education is more effective than the traditional form [9–12]. Other studies have shown that team-based learning and peer-assisted teaching can be effective as well [13, 14]. The necessity of a formal examination for the evaluation of basic surgical-skill, meanwhile, is universally recognized.

In the United States, a practical test for foreign medical students has been administered since 1998, and a clinical-skill examination has been a part of the United States Medical License Examination (USMLE) since 2004. The utility of these two examinations in the improvement of intern performance has been confirmed in two reports [15, 16]. In Korea, as noted above, clinical-skill tests have been part of the KMLE since 2009 [4]. These too have been found to improve the clinical performance of interns as well as residents. In fact, examinees have considered them to be helpful to primary medical care and residency training [17, 18].

Even after the introduction of tests of this type, students’ ability to perform basic surgical tasks such as surgical suturing still varies widely. Indeed, the implementation of basic surgical-skill education has been only imperfectly achieved, chiefly due to the difficulty of standardizing teaching and evaluation. For example, there are numerous intestinal-anastomotic methods and techniques, including continuous/interrupted, appositional/inverting/everting, Gambee, and Connell, among others [19–21]. Even in the case of simple suturing, almost all surgeons have their own preferences regarding, for example, the choice of suture material or the manner in which instruments are held. Of course, it is difficult to judge which preferences and which practices are the best. Current teaching programs are also problematic, as they are merely superficial and differ significantly from the actual practice. Medical students practice surgical suturing with a sponge for only a very limited amount of time, and this is why students often struggle with suturing in the real world despite having passed the KMLE.

The SSW program, a one-day intensive, simulation-based and motivational training course for medical students, was inaugurated in 2011. Its key aspects are in-depth training and application of simulation. The students are supplied with sponges as in the OSCE of the KMLE, after which they move on to simulation settings such as with SPs or operation-room simulation. In fact, there had been several differences among the tutors in terms of their preferences of suture style or scoring tendency in the initial assessment. Because of the differences, it was difficult to measure objectively the improvement in the students’ skills. In order to reduce inter-tutor variation, a procedural-standardization video clip was provided for tutors’ re-education prior to the program from 2015. Additionally, for the standardization of evaluation, check lists similar to those in the OSCE in the KMLE were developed. The initiatives for procedural and evaluative standardization had been undertaken in response to the confusion of earlier participants who had been instructed by tutors each bringing his or her own methods and tips to the program. The standardization of the tutors had a decisive effect on the program’s overall success.

We looked at the direct effect on each student’s surgical-suturing skill improvement. The median score and range were improved and decreased with statistical significance, respectively, after the program (median: 14 to 20, range: 6–20 to 17–20, *p < 0.000*, Table 2). These results showed that the individual variation in the surgical abilities of students had been reduced. Standardization of basic surgical-skill is essential if physicians with the minimum qualifications are to be reliably and consistently trained. Shapiro et al. reported that an effectively designed course-based undergraduate education, as compared with the traditional apprentice-based instruction, appeared to reduce the achievement gap between the highest performing students and the lesser-performing peers [22]. In fact, according to our results, low-year students showed more post-program improvement in basic surgical-skill than high-year students with statistical significance (Table 3). In the test before the program, low-year students obtained lower score than high-year students. However, after the program, the scores of the two groups were similar. While this may mean a greater increase of scores in low-year students, it may also mean there has been a standardization of the scores between the students.

**Fig. 2:**
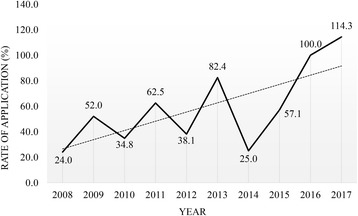
Trend of the rate of application to surgery over the last decade in eight institutions of the Catholic University of Korea

At the end of the program, we also surveyed on the students’ routes to participation, their opinions on useful or inadequate sessions, their overall levels of satisfaction, and their change of interest in a surgical career. Attracting competent junior medical students to a career in surgery, obviously, is crucial to any hospital’s short- and long-term viability. In this regard, it was promising that the survey results showed a positive post-program change in interest in a surgical career, specifically an increase from 51 to 74 students. This suggests that surgical-skill education programs such as SSW not only introduces the culture of surgery to medical students at an earlier time, but also helps to develop interest in medical students to pursue a career in surgery. Actually, rate of application to surgery in eight institutions of the Catholic University has increased from 24.0% in 2008 to 114.3% in 2017 (Fig. 2). We believe that because the SSW aimed to train medical students with the actual surgical technique, it gave the students confidence in performing the surgery themselves, and this confidence affected their decisions.

We collected additional data from previous participants who had attended the SSW program between 2011 and 2014. The data of the participants in 2015 and 2016 were not included because they are still students or interns. We sent each of the previous participants an e-mail and a short message that ask their job status now. Of 204 participants who had attended during the period, 132 (64.7%) participants replied. Among them, 24 (18.2%) participants were still medical students, 26 (19.7%) were interns, and 21 (15.9%) were in other fields such as military doctors or general physicians. Among 61 (46.2%) participants who are in residency now, 37 (60.7%) were surgeons and 24 (39.3%) were in the other departments. Among 37 surgeons, 9 (24.3%) were general surgeons and 28 (75.7%) were in the other surgical departments such as neurosurgery, thoracic and cardiac surgery, gynecology, orthopedics, plastic surgery, urology, and ear nose throat. The probability of becoming a surgeon was higher in the participants compared with a common medical student population.

Various other intensive surgical-skill education programs predate the present one. One, a “Surgical Skill Boot Camp,” targeted to students almost at intern status, convened for one week [23]. Several others are one-day intensive courses in plastic surgery and maxillofacial-surgical fields as career options [8, 24, 25]. This paper is, to our best knowledge, the first report of a program that is not only one-day intensive but also geared to the improvement and standardization of both surgical-skill and teaching methods, not to mention the promotion of general surgery as a career option for students.

## Conclusion

In summary, an intensive and standardized basic surgical-skill education program has been shown to have the potential to improve the suture-skill of medical students and stimulate their interest in surgery as a career option. More and better-standardized procedures and tutors and more systematic educational programs such as SSW such as SSW will be needed for medical students in the years to come.

## Additional files

Additional file 1: Raw data of SSW. Sheet 1 includes participants’ score before and after the program. Sheet 2 includes survey of the participants. (XLSX 22 kb)

## Abbreviations

CPX: Clinical performance examination
KMLE: Korean Medical License Examination
OSATS: Objective Structured Assessment of Technical Skill
OSCE: Objective-structured clinical examination
SNS: Social network services
SPs: Standardized patients
SSW: Surgical Skill Weekend
USMLE: United States Medical License Examination
